# The adaptive value of habitat preferences from a multi-scale spatial perspective: insights from marsh-nesting avian species

**DOI:** 10.7717/peerj.3164

**Published:** 2017-03-28

**Authors:** Jan Jedlikowski, Mattia Brambilla

**Affiliations:** 1 Faculty of Biology, Biological and Chemical Research Centre, University of Warsaw, Warsaw, Poland; 2 Settore Biodiversità e Aree protette, Fondazione Lombardia per l’Ambiente, Seveso (MB), Italy; 3 Sezione Zoologia dei Vertebrati, Museo delle Scienze, Trento, Italy

**Keywords:** Habitat selection, Adaptation, Spatial scale, Nest survival, Rallidae

## Abstract

**Background:**

Habitat selection and its adaptive outcomes are crucial features for animal life-history strategies. Nevertheless, congruence between habitat preferences and breeding success has been rarely demonstrated, which may result from the single-scale evaluation of animal choices. As habitat selection is a complex multi-scale process in many groups of animal species, investigating adaptiveness of habitat selection in a multi-scale framework is crucial. In this study, we explore whether habitat preferences acting at different spatial scales enhance the fitness of bird species, and check the appropriateness of single vs. multi-scale models. We expected that variables found to be more important for habitat selection at individual scale(s), would coherently play a major role in affecting nest survival at the same scale(s).

**Methods:**

We considered habitat preferences of two Rallidae species, little crake (*Zapornia parva*) and water rail (*Rallus aquaticus*), at three spatial scales (landscape, territory, and nest-site) and related them to nest survival. Single-scale versus multi-scale models (GLS and glmmPQL) were compared to check which model better described adaptiveness of habitat preferences. Consistency between the effect of variables on habitat selection and on nest survival was checked to investigate their adaptive value.

**Results:**

In both species, multi-scale models for nest survival were more supported than single-scale ones. In little crake, the multi-scale model indicated vegetation density and water depth at the territory scale, as well as vegetation height at nest-site scale, as the most important variables. The first two variables were among the most important for nest survival and habitat selection, and the coherent effects suggested the adaptive value of habitat preferences. In water rail, the multi-scale model of nest survival showed vegetation density at territory scale and extent of emergent vegetation within landscape scale as the most important ones, although we found a consistent effect with the habitat selection model (and hence evidence for adaptiveness) only for the former.

**Discussion:**

Our work suggests caution when interpreting adaptiveness of habitat preferences at a single spatial scale because such an approach may under- or over-estimate the importance of habitat factors. As an example, we found evidence only for a weak effect of water depth at territory scale on little crake nest survival; however, according to the multi-scale analysis, such effect turned out to be important and appeared highly adaptive. Therefore, multi-scale approaches to the study of adaptive explanations for habitat selection mechanisms should be promoted.

## Introduction

Choice of the breeding site may strongly affect survival rate and productivity of animal populations, and is thus commonly presumed to be an important driver of life-history evolution (e.g., for birds: Martin, 1995; Martin, 1998). Under natural selection, habitat preferences should therefore evolve to maximize animal fitness, i.e., animals are expected to select the best possible breeding sites which would allow for the highest reproductive outcome.

Although the selection of breeding site and the resulting habitat preferences are widely assumed to be adaptive (i.e., fitness is greater in preferred habitats), this assumption is rarely tested, and results are often equivocal (Arlt & Pärt, 2007; Mägi et al., 2009; Chalfoun & Schmidt, 2012). A review provided by Chalfoun & Schmidt (2012) showed that habitat preferences affected fitness outcomes (i.e., nest or seasonal reproductive success) in only 44% of analysed studies. The lack of congruence between habitat preferences and reproductive performance may occur for many different reasons, including inability to freely move between patches and locate optimal habitats (Forbes & Kaiser, 1994), asymmetric competition between species (Martin & Martin, 2001), difficulty in proper assessment of the actual qualities of environments by inexperienced individuals (Orians & Wittenberger, 1991), or by unpredictable variability in abiotic factors (Fletcher & Koford, 2004). In addition to the ecological reasons, many studies have reported examples of human-altered environments acting as ‘ecological traps’ in the habitat selection process (Schlaepfer, Runge & Sherman, 2002; Bonnington, Gaston & Evans, 2015).

Apart from the above ecological-evolutionary and anthropogenic explanations, sampling limitations and methodological issues can also result in an apparent lack of adaptiveness of habitat preferences. Small sample size may prevent the detection of relationships with habitat metrics affecting habitat choice and fitness outcomes (Battin & Lawler, 2006; Chalfoun & Schmidt, 2012). More importantly, habitat selection processes can operate at spatial and temporal dimension, and the adaptive value of such processes should be investigated at the appropriate scales. Habitat selection is generally approved to be a multi-scale spatial process, not only in birds (Brambilla, Rubolini & Guidali, 2006; Fuller, 2012), but also in other groups like insects (Krawchuk & Taylor, 2003), mammals (Chamberlain et al., 2003), reptiles (Harvey & Weatherhead, 2006), amphibians (Blomquist & Hunter, 2010), or fish (Brind’Amour et al., 2005). As a result, a proper investigation of adaptiveness of habitat-selection strategies requires framing analyses at the multiple spatial scales relevant for the study organism. However, the vast majority of studies have focused on the adaptive value of habitat preferences at a single spatial scale (Martin, 1998; Misenhelter & Rotenberry, 2000; Davis, 2005; Arlt & Pärt, 2007; Brambilla & Ficetola, 2012; Murray & Best, 2014; but see Chalfoun & Martin, 2007). When habitat selection is a multi-scale process, the lack of congruence or even mismatches between habitat preferences and fitness outcome may result from key habitat features that act at other scales and have been overlooked rather than from maladaptive choice or other ecological-evolutionary reasons.

Avian species qualify as an ideal model for the investigation of the adaptiveness of multi-scale habitat preferences. In birds, decisions related to the selection of the breeding site are usually determined by the availability of critical resources, such as food (Crampton & Sedinger, 2011; Barea, 2012) and refuge from predators (Forstmeier & Weiss, 2004; Forsman et al., 2013), which in turn may strongly affect multiple fitness outcomes. Occupying high-quality habitats determine, in general, higher fitness indices for clutches (nest survival, clutch size, clutch mass; Martin, 1998), fledglings (offspring mass and survival; Bloom et al., 2013; Germain et al., 2015), and adults (survival of individual birds, seasonal reproductive success, numbers of nesting attempts; Chalfoun & Martin, 2007). In response to patchiness and hierarchical structure of the environment, birds tend to recognize and select their habitat at several spatial scales (Fuller, 2012). As an example, for many passerine species the risk of predation is the primary force affecting habitat selection (Ibáñez-Álamo et al., 2015), and the choices performed at several scales are important mostly in terms of predation avoidance (Thompson, 2007). At the landscape scale, birds may try to select habitats with lower densities of predators, based on direct cues, i.e., avoiding predators once they are detected (Cresswell, 2008; Dinkins et al., 2014), or using proximate cues, e.g., by avoiding habitats with higher risk of predation such as smaller and more isolated patches (Dolman, 2012). At the territory and nest-site scale, factors such as vegetation density and foliage cover, as well as a number of potential nest sites within territory plot, are commonly thought to be crucial factors that hamper predator search efforts and reduce probability of nest predation (Martin & Roper, 1988; Martin, 1993; Chalfoun & Martin, 2009). Finally, different types of social and public information, such as information about breeding success of neighbours, may be collected by birds at particular scales to obtain knowledge about risk of nest predation (Lima, 2009). Therefore, for species that assess habitat suitability within multiple spatial scales, the overall adaptive value of habitat preferences should be clearly evaluated at several ecologically relevant spatial scales.

In this study, we evaluated the effect of scale on the adaptiveness of two bird species by analysing nest survival and its relation to habitat preferences at three spatial scales—landscape, territory, and nest-site. We used nest survival as a relevant and widely measured fitness component to check congruence with evolved habitat preferences. Our objectives were (1) to investigate the relative effect of factors acting at different spatial scales on nest survival, (2) to investigate factors affecting nest survival in a multi-scale context and to check whether the effects from single-scale analyses can be relevant also when considering all spatial scales at once, and (3) to evaluate the adaptiveness of multi-scale habitat preferences, by considering the overall effect of habitat suitability and consistency between the effect of variables on habitat selection and on nest survival. We explored the potential effect on nest survival of habitat factors acting at different spatial scales and found to be important (selected by multi-scale models) or potentially important (apparently important only according to single-scale analyses) for habitat selection in the study species. We expected that factors found to be more important for habitat selection at individual scale(s), would coherently play a similar role in affecting nest survival at the same scale(s).

## Materials & Methods

### Study system

We focused on two bird species belonging to the Rallidae family, little crake (*Zapornia parva*; formerly *Porzana* genus) and water rail (*Rallus aquaticus*). These two migratory species occupy a wide range of aquatic habitats with still or slow-moving water (Taylor & Van Perlo, 1998). They require tall, dense emergent vegetation as nesting sites and protection against predators. The ones found in the study area are marsh harrier (*Circus aeruginosus*), raccoon dog (*Nyctereutes procyonoides*), least weasel (*Mustela nivalis*), and American mink (*Neovison vison*; Jedlikowski, Brzeziński & Chibowski, 2015; J Jedlikowski, pers. comm., 2011). Previous studies showed that habitat selection is a multi-scale process in these species and that rallid occurrence was mostly affected by habitat factors at the territory scale (Jedlikowski et al., 2016). Here, we use the same study area and habitat factors measured for the habitat selection process to evaluate adaptiveness of the habitat preferences.

The two species were surveyed at 30 small water bodies located between 53°47′–53°53′N and 21°33′–21°47′E, in the central part of the Masurian Lakeland (north-eastern Poland). The area of these water bodies varied from 0.2 to 10.0 ha (mean 2.21), water level fluctuated seasonally but the maximum depth did not exceed 1.5 m, and water pH ranged from 6.3 to 8.2. All ponds were located in natural depressions filled with organic sediments and were overgrown mainly by bulrush (*Typha* spp.), common reed (*Phragmites australis*), sedges (*Carex* spp.), and bushes of grey willow (*Salix cinerea*). These inland water bodies were surrounded by a mosaic of wetland-agricultural landscapes including arable fields, meadows, pastures, sparse rural areas, forests and other wetland complexes (lakes, marshes, and ditches; see Jedlikowski et al., 2016).

The study was purely observational with no manipulation of animals. The study fulfilled the current Polish Law, and the relevant committee, Regional Directorate for Environmental Protection, allows us to carry out this research (approval number WOPN-OOP.6402.45.2012.AWK).

### Nest monitoring

For both little crake and water rail, which are precocial species, the nesting period is critical for successful breeding, so we used nest survival as a fitness component. To obtain nest fate of both species, we collected data from late-April to early-August over three successive breeding seasons (2012–2014). We used call-playback surveys to identify territories occupied by studied species within each season (Jedlikowski, Brambilla & Suska-Malawska, 2014). Subsequently, we systematically searched within patches of littoral vegetation for nests of little crake and water rail, and marked the position of each nest using a handheld GPS receiver (GRS-1, Topcon; accuracy less than 0.5 m). We monitored each nest beginning two days after finding it to assess the stage of the clutch (egg laying, incubating or hatching stage). Afterwards, we checked each nest at 5–7 day intervals until the clutch successfully hatched or was found depredated. We determined nest success when at least one chick was observed (or heard) in or in close proximity to the nest (<2 m), and no traces of nest predation were detected. Crushed pieces of shells with remains of yolk found in the nest, or close by in the water, were considered as evidence of nest predation. Also partially depredated clutches were treated as depredated, as such nests were always abandoned by adults. We also considered nest loss when we found empty nests accompanied by damage of nest material and surrounding vegetation during the presumed incubation period.

### Habitat measurements

We used circular plots centred on each nest to measure habitat characteristic and composition of vegetation around nests at three spatial scales: ‘landscape’ (200-m radius), ‘territory’ (14-m radius), and ‘nest-site’ (3-m radius). Those scales were chosen according to the previous habitat selection studies with little crake and water rail. The extent of 200-m radius was associated with the best performing landscape-scale habitat selection model for both rallids (Jedlikowski et al., 2016). This radius was consistent with other studies that assessed relationship between Rallidae species and landscape features (e.g., Austin & Buhl, 2013; Glisson et al., 2015). Territory and nest-site scale radii were selected based on telemetry study (Jedlikowski, Brambilla & Suska-Malawska, 2014; Jedlikowski & Brambilla, 2017). Within each scale, we recorded habitat variables deemed as potentially important (i.e., included in the most supported models at each scale) by the habitat selection study (see Jedlikowski et al., 2016 and Table 1).

To describe habitat characteristics at the landscape scale, we used spatial analyst tools in ArcGIS 10.2 (ESRI, 2013) to measure the extent and configuration of the main land cover classes (see Table 1). Land cover classes were assigned based on aerial photos of water bodies and surrounding habitats taken in April 2012 and ground-truthed each year in the field. Each homogeneous habitat patch was mapped when one of its dimensions exceeded 10 m. In the case of territory scale (represented by a 14-m radius plot around nest), mean vegetation density and water depth were measured, and extent of emergent plant cover was estimated using ArcGIS 10.2 (see Table 1). To estimate the percentage cover of emergent vegetation species at the territory scale, patches of vegetation were mapped when one of their dimensions exceeded 2 m and classified according to the main plant species. Patches were mapped by walking through and recording position by means of a handheld GPS receiver. When nests were situated closer than 14 m to the water-land edge, and territory plots extended to the terrestrial habitat (space not used by birds), the border of potential territories was adjusted to the water body shoreline (in 70% cases for little crake and 76% cases for water rail). As a result, in such cases, all measurements were taken within the adjusted territory plot, in the same way as in the habitat selection study for these species (see Jedlikowski et al., 2016). All the territory measurements were performed immediately after clutch hatched or was depredated, to avoid vegetation damage or disturbance to birds during the nesting period. At the nest-site scale, all measured habitat characteristics concerned nest position within littoral vegetation and the structure of vegetation in the immediate vicinity of the nest, and were taken on the same day that the nest was found (see Table 1).

**Table 1:** Description of habitat characteristics measured around nests of little crake and water rail within three spatial scales (landscape, territory and nest-site), that were found to be important in breeding site choice (c.f. Jedlikowski et al., 2016) and used in the analyses of the nest survival model of both rallids in the Masurian Lakeland, Poland.

Variable	Acronym	Description
**Landscape scale**		
Arable land^a^	arable.l	Extent of cultivated fields (%)
Urbanised area^a^	urban.a	Extent of artificial surface: urban fabric, industrial units, and road network (%)
Emergent vegetation^a^^,^^b^	em.veg	Extent of water bodies overgrown by emergent vegetation (%)
Woody vegetation^b^	wood.veg	Extent of water bodies overgrown by shrub and trees (%)
Water body shape^a^	wat.shape	Perimeter-area ratio index (Helzer & Jelinski, 1999) estimated by dividing total water body perimeter by total water body area (m/ha)
Water body fragmentation^a^^,^^b^	wat.frag	Proximity index (*PX*) (Gustafson & Parker, 1994) calculated for *n* water bodies as $PX={\mathop{\sum }\nolimits }_{i=1}^{n} \left( {S}_{i}/{z}_{i} \right) $, where *S*_*i*_ was the area of each water body within a particular buffer (even those which partially lie within the radius) and *z*_*i*_ the shortest linear distance to an adjacent water body (ha/m)
**Territory scale**		
Reed cover^a^^,^^b^	reed.cov	Extent of *Phragmites australis* cover (%)
Sedges cover^b^	sed.cov	Extent of *Carex* spp. cover (%)
Willow cover^a^	will.cov	Extent of *Salix* spp. cover (%)
Open water^b^	op.wat.t	Extent of open water surface (%)
Vegetation density^a^^,^^b^	veg.dens	Mean vegetation density within territory plot (five measurements taken every 2 m in each cardinal direction around nest; measurements taken outside the water bodies were removed from further analysis). Vegetation density was estimated as the percentage cover of vegetation within a 0.5 × 0.5 m quadrant (estimated with 10% precision each time by the same investigator)
Water depth^a^^,^^b^	wat.dep.t	Mean water depth within a territory plot (five measurements taken every 2 m in each cardinal direction around nest; measurements taken outside the water bodies were removed from further analysis) (cm)
**Nest-site scale**		
Plant species^a^	plant.sp	Species of emergent plants at nest site
Vegetation stage^a^^,^^b^	veg.st	Stage of emergent vegetation at nest site (1 = fresh, 2 = previous years, 3 = mixed)
Vegetation cover^b^	veg.cov	Percentage cover of emergent vegetation within a 3-m radius plot around nest (estimated with 10% precision each time by the same investigator)
Vegetation height^a^^,^^b^	veg.ht	Mean height of emergent vegetation within nest plot (measurements taken from the water surface from ten random points around nest) (cm)
Water depth^a^^,^^b^	wat.dep.n	Mean water depth in a 3-m radius plot around nest calculated from ten random measurements within the plot (cm)

**Notes.**

a Variable importance in single-scale models of habitat selection for little crake.

b Variables important for water rail.

### Data analysis

In our study system, all nest data had a strong spatial structure, because of pond distribution in the landscape and of nest distribution within ponds. This prevented us from using standard nest-survival methods based on generalized linear models (Dinsmore, White & Knopf, 2002; Hazler, 2004), which do not allow for a proper treatment of spatial autocorrelation. Therefore, we performed a two-step analysis to assess the relationship between habitat preferences and nest survival using methods suited to deal with spatially autocorrelated data. Before running models, all variables were standardized, in order to compare the relative effects (Schielzeth, 2010) and better evaluate multicollinearity problems (Cade, 2015).

For the first step, we used generalized least squares (GLS) models. This regression technique allows for the incorporation of the spatial structure into the model’s error and is considered among the best methods to deal with spatially autocorrelated datasets (Dormann et al., 2007; Beale et al., 2010). We built species-specific GLS models with the number of days a nest survived as the dependent variable, and year, initiation date and habitat factors as covariates. In precocial species, such as little crake and water rail, it is known that older nests, i.e., those that have survived more days, have higher survival rates than younger nests because nests located in more risky sites will be depredated in early stages (Klett & Johnson, 1982; Dinsmore, White & Knopf, 2002). In our analysis, we used only nests found during the egg laying period, which allowed us to correctly estimate the number of survived days for each nest, from the nest initiation day (the day when the first egg was laid) to the day when chicks hatched or clutch was depredated. For clutches found after initiation day, we used the backdating method to estimate the first-egg date, assuming that the birds lay one egg per day (Taylor & Van Perlo, 1998). When a nest failed between two consecutive visits, we estimated time of failure as the mid-point between visits. Finally, we adjusted the number of days a successful nests survived to 23 days for little crake and 27 days for water rail. All nests which survived to this age were successful, but some nests required a few more days for hatching (in little crake 27 nests required one or two days more; in water rail 10 nests were successful after one or two days more, and four nests after three to four days more, respectively). By this adjustment, we avoided giving an excessive weight within models to successful nests that required extra time to hatch. We used an information-theoretic approach (Burnham & Anderson, 2002), ranking all possible models according to the Akaike Information Criterion corrected for small sample sizes (AIC_C_). Given that initiation time and year variation had been reported several times among the main factors affecting nest success in several bird species (e.g., Mazgajski, 2002; Dinsmore & Dinsmore, 2007), they were kept as fixed factors in the models. In fact, it is known that shifts in predator communities or in the availability of alternative prey for predators, as well as climatic conditions, may affect nest success within a breeding season and over different years (e.g., Moynahan et al., 2007; Shitikov, Fedotova & Gagieva, 2012). For each model, we checked the potential occurrence of multi-collinearity among predictors according to the relative values of the VIF (Variance Inflation Factor). All variables tested in the models had VIF <  3, thus multi-collinearity was not an issue for the analyses. Therefore, we ranked models according to the relative AIC_C_-values for each species and at each spatial scale. Then, we considered the most supported models (ΔAIC_C_ <  2) and excluded those with uninformative parameters (i.e., models comprising a more parsimonious model as nested, plus some additional factors; Arnold, 2010). Finally, we carried out model averaging (full averaging) among the remaining models, or took the most parsimonious model when no others remained.

The above procedure was adopted for all the analyses required by the three objectives of the study. For objective 1, we carried out the analysis at each spatial scale (landscape, territory, nest-site) for each species. For objective 2, we used a multi-scale model for each species. After species-specific model ranking for each spatial scale, we chose for each species all the variables comprised in the most parsimonious models (models with ΔAIC_C_ <  2). With the resulting sets of variables, we worked out multi-scale models for each species, ranking all possible models according to AIC_C_ (matching the approach adopted in the habitat selection study, Jedlikowski et al., 2016). For objective 3, we used the estimated habitat suitability as a predictor (occurrence probability as calculated according to the species-specific multi-scale models of habitat selection, reported in Table 5 in Jedlikowski et al., 2016), to assess whether multi-scale habitat preferences were adaptive or not. If habitat preferences are adaptive, the predicted habitat suitability should predict nest survival. In all the final models, residuals approached a normal distribution.

As the second step, after running GLS models, we checked for consistency of the effects of the variables affecting the number of days a nest survived in relation to the final fate of nests, by taking into account nest exposure. Therefore, we performed a further analysis, implementing a binomial model with a logit link function, with the binomial numerator being 0 or 1 (failure or success) and the binomial denominator being number of days a nest survived (cf. Mayfield logistic regression; Hazler, 2004). We performed this analysis by means of spatial Generalized Linear Mixed Models via Penalized Quasi-Likelihood (glmmPQL), assuming a binomial error distribution, relating variables selected by GLS models to nest success/failure. glmmPQL allows modelling spatial data with non-normal distribution and is considered among the best techniques to handle this type of data (Dormann et al., 2007).

For GLS and glmmPQL models, we adopted a Gaussian spatial correlation structure, but models with different structures (spherical, exponential) led to the same or very similar results. All analyses were performed using the software R (R Development Core Team, 2013) and the packages ‘MuMIn’, ‘mass’ and ‘nlme’ (Venables & Ripley, 2002; Pinheiro, Bates & Debroy, 2010; Bartoń, 2014).

Finally, habitat preferences (objective 3) were considered as adaptive when: (i) habitat suitability as calculated with the multi-scale models for habitat selection significantly predicted variation in nest survival, with a positive effect (we used the logit value for the regression analyses); (ii) variables importantly affecting habitat selection (included in the multi-scale models, Table 5 in Jedlikowski et al., 2016) showed the same kind of effect on both habitat preference and nest survival.

## Results

Little crake chicks hatched in 32 out of 51 monitored nests (63%), and water rail chicks hatched in 19 out of 50 nests (38%). Descriptive statistics of all habitat parameters measured in successful and predated nests of water rail and little crake are shown in Supplemental Information 1.

### Factors affecting nest survival based on individual and the multi-scale approach

At the landscape scale, the most parsimonious GLS model for little crake included only fixed factors (year and initiation; Table 2). For water rail, nest survival was negatively affected by the extent of emergent vegetation (β =  − 2.68, *SE* = 1.16, *p* = 0.025; Table 2). At the territory scale, the top-ranked models indicated that vegetation density was positively associated with nest survival in both species (β = 5.02, *SE* = 0.66, *p* < 0.001 for little crake; β = 5.19, *SE* = 1.02, *p* < 0.001 for water rail; Table 2). At the nest-site scale, the nest survival of rallid nests was mostly affected by a positive relationship with vegetation height (β = 3.61, *SE* = 0.93, *p* < 0.001 for little crake; β = 3.39, *SE* = 1.16, *p* = 0.005 for water rail; Table 2).

**Table 2:** Summary of the GLS models describing nest survival of little crake and water rail nests at the three spatial scales. Models are ranked according to Akaike’s information criterion corrected for small sample size (AIC_C_), the difference in AIC_C_ from the best supported model (ΔAIC_C_), Akaike’s weights (*w*_*i*_), and −2 log-likelihood values (logLik). Only models with ΔAIC_C_ <  2 are shown. Negative (−) or positive (+) relationships were shown between variables and nest survival rate for little crake and water rail. Year and initiation day (‘intt’) were treated as fixed variables. For the rest of variable acronyms, see Table 1.

Model	*df*	logLik	AIC_C_	ΔAIC_C_	*w*_*i*_
**Little crake**					
Landscape scale					
Year (−) + intt (−)	6	−168.93	351.8	0.00	0.31
Year (−) + intt (−) + arable.l (−)	7	−167.91	352.4	0.66	0.22
Territory scale					
Year (−) + intt (−) + veg.dens (+)	7	−148.26	313.1	0.00	0.45
Year (−) + intt (−) + veg.dens (+) + wat.dep.t (+)	8	−147.56	314.6	1.44	0.22
Nest-site scale					
Year (−) + intt (−) + veg.ht (+)	7	−161.84	340.3	0.00	0.48
Year (−) + intt (−) + veg.ht (+) + wat.dep.n (+)	8	−160.68	340.8	0.49	0.37
**Water rail**					
Landscape scale					
Year (−) + intt (−) + em.veg (−)	7	−172.10	360.9	0.00	0.40
Territory scale					
Year (−) + intt (−) + veg.dens (+) + reed.cov (+) + op.wat.t (+)	9	−161.30	345.1	0.00	0.29
Year (−) + intt (−) + veg.dens (+) + reed.cov (+)	8	−162.87	345.2	0.14	0.27
Year (−) + intt (−) + veg.dens (+) + op.wat.t (+)	8	−163.50	346.5	1.40	0.14
year (−) + intt (−) + veg.dens (+)	7	−164.96	346.6	1.48	0.14
Nest-site scale					
Year (−) + intt (−) + veg.ht (+) + veg.cov (+)	8	−168.43	356.4	0.00	0.39
Year (−) + intt (−) + veg.ht (+)	7	−170.43	357.5	1.16	0.22

The multi-scale models (i.e., the ones including the most relevant factors from different scales; see Tables 3 and 4) showed the positive and prevalent effect of vegetation density within territory scale on nest survival in both species. Moreover, nest survival was positively affected by water depth within territory scale and vegetation height at nest-site scale in little crake, as well as negatively related to emergent vegetation extent at the landscape scale in water rail. A positive effect on nest survival for water rail was found also for vegetation height and reed cover, which were, however, characterized by lower relative importance.

**Table 3:** Multi-scale model describing nest survival in little crake and water rail: the most parsimonious GLS models (ΔAIC_C_ < 2) for each species are shown. Negative (−) or positive (+) relationships were shown between variables and nest survival rate for both rallids. Year and initiation day (‘intt’) were treated as fixed variables. For the rest of variable acronyms, see Table 1.

Model	*df*	logLik	AIC _C_	ΔAIC _C_	*w*_*i*_
**Little crake**					
Year (−) + intt (−) + veg.dens (+) + wat.dep.t (+) + veg.ht (+)	9	−140.53	303.4	0.00	0.46
Year (−) + intt (−) + veg.dens (+) + wat.dep.t (+) + veg.ht (+) + arable.l (−)	10	−139.91	305.3	1.87	0.18
**Water rail**					
Year (−) + intt (−) + em.veg (−) + veg.dens (+) + veg.ht (+)	9	−152.59	327.7	0.00	0.33
Year (−) + intt (−) + em.veg (−) + veg.dens (+) + veg.ht (+) + reed.cov (+)	10	−151.59	328.8	1.15	0.19
Year (−) + intt (−) + em.veg (−) + veg.dens (+) + reed.cov (+)	9	−153.18	328.9	1.18	0.18
Year (−) + intt (−) + em.veg (−) + veg.dens (+) + veg.ht (+) + op.wat.t (+)	10	−152.01	329.7	1.98	0.12

**Table 4:** Model averaged parameter (based on models with ΔAIC_C_ < 2) and relative variable importance (measured considering the sum of the Akaike weights over all models in which that variable appears) of predictors from multi-scale models of nest survival for water rail and the most parsimonious model for little crake. In all models year (2012) was used as the reference category. In water rail, covariates are ranked according to cumulative weights. For variable acronyms, see Table 1.

Variable	β	SE	∑*w*_*i*_	*P*
**Little crake**				
Intercept	19.41	1.38		
Year (2013)	−2.42	1.54		0.124
Year (2014)	−0.22	2.02		0.912
Initiation day	−1.41	0.66		0.040
veg.dens	4.16	0.61		<0.001
veg.ht	3.00	0.71		<0.001
wat.dep.t	2.02	0.68		0.005
**Water rail**				
Intercept	19.32	1.75		
Year (2013)	1.51	2.47	1.00	0.552
Year (2014)	−3.79	2.17	1.00	0.089
Initiation day	−0.71	1.15	1.00	0.540
em.veg	−3.40	0.85	1.00	<0.001
veg.dens	4.89	0.84	1.00	<0.001
veg.ht	1.93	1.60	0.64	0.230
reed.cov	0.97	1.40	0.36	0.489

The analysis of nest fate (success/predation) in relation to the number of days a nest survived based on glmmPQL models showed that variables affecting nest survival (expressed as a number of days) had the same effect (positive or negative) on nest fate expressed as a binomial outcome (see Supplemental Information 2).

### Comparing models’ performance across scales

For both species, AIC_C_ values suggested the following hierarchy of models, ranked from most to least supported ones: territory, nest-site and landscape scales (see Table 2). This pattern was suggested by the amount of variation explained by models at each single scale: the *R*^2^ of territory scale models were equal to 0.59 for little crake and to 0.53 for water rail, whereas *R*^2^ for nest-site scale models was 0.30 and 0.38, respectively, and for landscape scale models was 0.07 and 0.28, respectively. Compared to the top-ranked single-scale models, multi-scale models were more parsimonious for both species and explained a higher amount of variation: the *R*^2^ of multi-scale models was 0.70 for little crake and 0.67 for water rail (considering the most parsimonious model for computation).

### The adaptive value of multi-scale habitat preferences

Habitat suitability was strongly correlated with nest survival both in little crake (β = 1.35, *SE* = 0.27, *p* < 0.001) and in water rail (β = 0.07, *SE* = 0.03, *p* = 0.014), suggesting that multi-scale habitat preferences were adaptive in both species.

**Figure 1:**
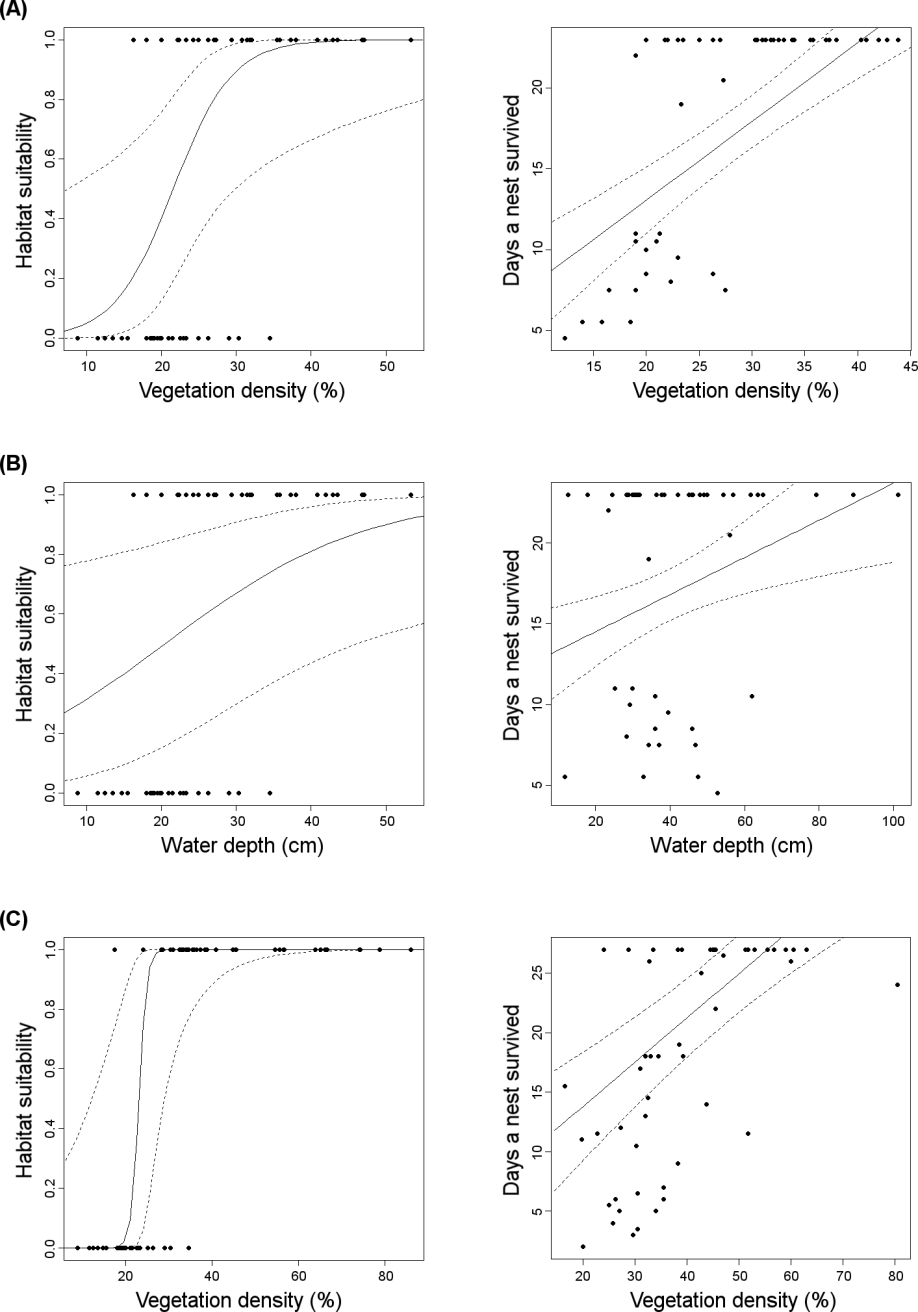
Graphical representation (solid line: predicted values; dashed line: 95% confidence intervals) of adaptive habitat preferences in relation to nest survival in rallids. Little crake selected territories with denser vegetation (A) and deeper water (B), which increased nest survival. Water rail preferred territories with denser vegetation that positively affected nest survival rate (C). For habitat suitability, dots represent nest occurrence (value 1) or absence (value 0; data derived from Jedlikowski et al., 2016). For nest survival, each dot represents a rallid nest; nests that survived at least 23 days were successful for little crake; nests that survived at least 27 days were successful for water rail.

Vegetation density and mean water depth at the territory scale were positively selected by little crake during habitat selection and were the main factors affecting nest survival (Figs. 1A, 1B). This suggests a strong adaptive value of habitat choice. In water rail, vegetation density at the territory scale was positively associated with both occurrence and nest survival, again suggesting adaptiveness of the preference for sites with denser vegetation (Fig. 1C).

## Discussion

### The importance of multi-scale approaches in the study of bird breeding ecology

Habitat selection has been increasingly regarded as a multi-scale process, encompassing different spatial scales in a wide range of species (McGarigal et al., 2016). Despite the increasingly clear importance of multi-scale approaches to habitat selection, very few previous studies have simultaneously evaluated the fitness outcomes of habitat choice at multiple scales (e.g., Chalfoun & Martin, 2007; Brambilla et al., 2010). This has generally prevented an adequate assessment of the adaptive habitat preferences for species that perform their decisions at several spatial scales.

Our results provide an evidence for the adaptive value of multi-scale habitat preferences, as demonstrated by the strong positive effect of habitat suitability on nest survival. Furthermore, our work provides evidence for a congruent effect of factors acting across multiple spatial scales over both habitat selection and reproductive outcomes. In particular, the most important habitat factor driving site selection in both rallids is vegetation density at the territory scale (Jedlikowski et al., 2016), the factor that most affected nest survival rate in both species (Tables 3 and 4). An effect was found also for water depth at the territory scale for little crake. Therefore, the habitat features important in habitat selection at the territory scale, clearly affected nest fate in the studied species. In general, such results strongly point towards an adaptive value of multi-scale habitat preferences in both species, and further demonstrate the overwhelming importance of the territory scale in the breeding ecology of little crake and water rail (cf. Jedlikowski et al., 2016). It is possible that other habitat characteristics that were selected or avoided in this study but did not affect nest survival may have had consequences for other fitness components (e.g., post-fledging survival, adult survival, lifetime reproductive success). In fact, multiple environmental factors across multiple spatial scales may optimize fitness via different habitat selection strategies (Chalfoun & Martin, 2007).

**Figure 2:**
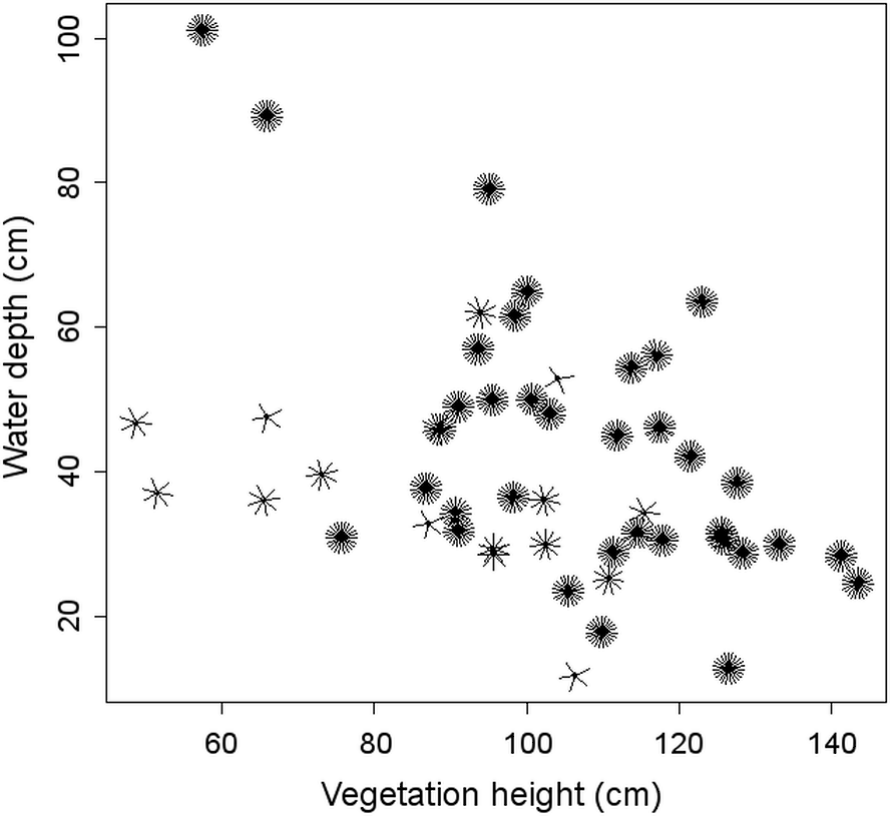
Sunflower plot representing little crake nest survival in relation to mean water depth at territory scale and vegetation height at nest-site scale. Each sunflower is an individual nest, and the number of ‘petals’ is the number of days survived by the nest.

Assessing adaptiveness at single spatial scales instead of multiple scales may lead to contrasting evaluations because of missing critical features belonging to other not considered scales. As an example, within our study systems, water depth had only a weak effect at the territory scale on little crake nest survival; however, according to the multi-scale model, water depth was important and the preference for deeper sites in the multi-scale habitat selection process appeared highly adaptive. The much greater importance of water depth at the multi-scale model than at the single territory scale, which can appear rather counterintuitive at first glance, is easily explained by the combined effect displayed by mean water depth at the territory scale and vegetation height at nest-site scale (Fig. 2). Nests placed in sites with tall vegetation were mostly successful, irrespectively of water depth, whereas nests hidden behind short vegetation survived longer only when they were surrounded by deeper water. This finding suggests the importance of evaluating adaptiveness considering relevant factors belonging to multiple spatial scales. The opposite pattern was found for emergent vegetation at the landscape scale: it was positively selected by water rail according to the single-scale model (Jedlikowski et al., 2016) but had a negative impact on nest survival (Tables 3 and 4). This would be regarded as maladaptive habitat choice; however, the multi-scale model of habitat selection for water rail suggested that the true determinants of species occurrence are different variables, and that emergent vegetation may be just a cue for the availability of suitable habitats at finer scales (Jedlikowski et al., 2016). This kind of partial evidence for adaptiveness, which arises when looking at single spatial scales but disappears when considered within a multi-scale context, suggests additional caution in evaluating the adaptive value of habitat preferences using single scale approaches. These are likely to overestimate the importance of minor determinants of habitat selection and/or reproductive output.

### The varying impact of different spatial scales on nest predation risk

Our results showed that both in little crake and water rail the reduction of nest predation risk was mostly determined by fine-scale habitat preferences at territory and nest-site scales. This was consistent with Chalfoun & Martin (2007), which found that habitat preferences at smaller spatial scales, but not at broader-landscape ones, reduced nest predation risk of Brewer’s sparrow (*Spizella breweri*). It seems that habitat selection at the landscape scale could be more relevant for nestling survival (Chalfoun & Martin, 2007), a fitness component which was not included in our study. At the landscape scale birds may focus on selecting areas rich in food, which may facilitate feeding of nestlings, increasing their mass and survival (Chalfoun & Martin, 2007). On the other hand, determinants of occurrence at smaller spatial scales may be related to particular habitat structure selected to reduce predation risk (Martin, 1998). Such a pattern was found in mallards (*Anas platyrhynchos*), where duckling survival was linked to selection of wetlands by brood-rearing adults at the landscape scale, but not to local-scale preferences (Bloom et al., 2013). Nevertheless, the mechanisms that link survival of chicks to landscape scale attributes have to be further investigated.

The preeminent role of territory choice in reducing predation risk is in disagreement with the conceptual model proposed by Thompson et al. (2002), which tried to explain spatial and temporal variability in nest predation. Thompson et al. (2002) predicted that larger scale factors should be more important determinants of nest survival as they provide context or constraints for smaller scale effects. However, according to the explanatory power of the single-scale models, the most relevant scale for both rallids was the territory, followed by nest-site, whereas landscape scale was the least influential. The crucial role of proper territory settlement has been also found in other marsh nesting species. For example, survival of artificial nests located within reed bunting (*Emberiza schoeniclus*) territories was higher compared to the nests placed in non-territories that were mostly predated by marsh harrier (Trnka, Peterková & Grujbárová, 2011). In the case of territorial species, such as little crake and water rail, which have to find and protect all the required resources within very limited areas, the proper choice of high-quality territories may affect not only reproductive success, but also determine individual fitness, mating success, and survival of adult birds (Best, 1977; Currie, Thompson & Burke, 2000; Przybylo, Wiggins & Merila, 2001).

### Adaptiveness of habitat selection in rallids

In little crake and water rail, the basic determinants of habitat selection were also the main factors affecting nest survival. The adaptive selection of dense vegetation stands is easily explained by considering the main nest predator of both species, the marsh harrier (Jedlikowski, Brzeziński & Chibowski, 2015). Nests placed within denser vegetation were obviously more difficult to find by this raptor, which searches for its prey by flying over wetlands. Greater nest concealment was also found to increase nest survival of other marsh-nesting species exposed to marsh harrier predation, such as great bittern (*Botaurus stellaris*; Polak, 2007) or common pochard (*Aythya ferina*; Albrecht et al., 2006). All these results are in agreement with the nest-concealment hypothesis, which suggests that high vegetation density may, on the one hand, impede the ability of predators and brood parasites to locate and access nests, and, on the other hand, may reduce the visual, olfactory, or auditory cues emitted by potential prey (Martin, 1993; Borgmann & Conway, 2015). Further, denser vegetation may contain more potential nest sites that must be searched by predators, thus further reducing the probability of predation as predators may give up before finding the occupied site (potential-prey-site hypothesis; Martin, 1993). Predators may not search vegetation randomly, but look for specific habitat features to locate prey (Martin & Roper, 1988; Chalfoun & Martin, 2009). Recent experimental studies indeed showed that nest concealment did not itself increase nest survival, but occupying sites with more potential nest sites was the mechanism that significantly influenced nest predation risk (Chalfoun & Martin, 2007; Chalfoun & Martin, 2009). However, the survival rate of both rallids was not related to the extent of emergent vegetation within territory scale. Furthermore, the fate of water rail nests was even negatively affected by the emergent vegetation cover at the landscape scale. Therefore, in both species, the selection of dense vegetation structure seems to be directly related to minimizing the probability of nest predation (confirming nest-concealment hypothesis rather than potential-prey-site hypothesis).

In little crake, which prefers sites with deeper water level than water rail (Jedlikowski, Brambilla & Suska-Malawska, 2014), water depth was an important driver for nest survival. Water depth has been repeatedly reported as a crucial factor reducing predation of nests situated at deeper sites (e.g., Hoover, 2006). However, because the main nest predator, i.e., the marsh harrier, should not be affected by water depth, such preferences may result from an anti-predator strategy towards potential mammalian predators. In particular, water depth at the territory scale seemed especially relevant in relation to vegetation height at the nest-site scale, as discussed above.

## Conclusions

Our study has provided evidence for adaptiveness of multi-scale habitat preferences in two bird species, highlighting the greatest importance of the territory scale for nest survival and habitat selection process. Our results demonstrate how single-scale models may be inadequate to investigate the adaptive value of habitat preferences, potentially revealing apparent or partial adaptiveness. On the other side, multi-scale assessments may help depict a thorough picture of adaptiveness, revealing, for example, the concomitant effects of factors operating at different spatial scales. Therefore, multi-scale approaches to the study of adaptive explanations for habitat selection mechanisms should be promoted.

##  Supplemental Information

10.7717/peerj.3164/supp-1 Supplemental Information 1 Descriptive statistics of environmental variables measured at successful and depredated nests of little crake and water rail

10.7717/peerj.3164/supp-2 Supplemental Information 2 Multi-scale glmmPQL models describing the effect of variables selected by GLS models on nest fate

10.7717/peerj.3164/supp-3 Data S1 Raw data Nest survival and habitat parameters database. Nest coordinates were not included for conservation reasons.

## Additional Information and Declarations

### Field Study Permissions

The following information was supplied relating to field study approvals (i.e., approving body and any reference numbers): The study fulfilled the current Polish Law and the relevant committee, Regional Directorate for Environmental Protection, approved this research (approval number WOPN-OOP.6402.45.2012.AWK).

### Data Availability

The following information was supplied regarding data availability: The raw data has been supplied as a Supplementary File.
