# Supplementary material for: The adaptive value of habitat preferences from a multi-scale spatial perspective: insights from marsh-nesting avian species
Source: PeerJ. 2017 Mar 28;5:e3164. doi: 10.7717/peerj.3164 (PMC5372843; doi:10.7717/peerj.3164)
Supplement: Supplemental Information 1 [file peerj-05-3164-s001.pdf]

Table S1. Descriptive statistics of environmental variables measured at successful and depredated nests of little crake (mean ± se). For categorical variables (vegetation species, vegetation stage) the percentage of nests in each category is shown. In vegetation species only two most common species are included.

Variable	Successful nests (n = 32)	Depredated nests (n = 19)
**Landscape scale**		
Arable land (%)	1.85 ± 0.98	5.01 ± 2.95
Urbanized area (%)	0.32 ± 0.14	0.35 ± 0.21
Emergent vegetation (%)	14.77 ± 0.77	15.43 ± 1.82
Woodie vegetation (%)	2.28 ± 0.59	2.01 ± 0.40
Water body shape (m/ha)	468.1 ± 27.8	484.6 ± 35.8
Water body fragmentation (ha/m)	0.141 ± 0.013	0.145 ± 0.023
**Territory scale**		
Reed cover (%)	5.00 ± 2.17	2.22 ± 1.43
Sedges cover (%)	6.57 ± 2.64	4.90 ± 2.42
Willow cover (%)	1.96 ± 1.17	0.00 ± 0.00
Open water(%)	29.06 ± 3.89	30.89 ± 5.14
Vegetation density (%)	32.73 ± 1.26	20.36 ± 1.00
Water depth (cm)	43.93 ± 3.61	37.41 ± 2.99
**Nest-site scale**		
Vegetation species (*Typha* spp.)	78.1%	68.4%
Vegetation species (*Carex* spp.)	15.6%	26.3%
Vegetation stage (previous years)	34.4%	31.6%
Vegetation stage (mixed)[c]	62.5%	68.4%
Vegetation height (cm)	107.3 ± 3.6	90.5 ± 4.7
Vegetation cover (%)	46.56 ± 1.87	47.89 ± 3.27
Water depth (cm)	42.83 ± 3.72	42.70 ± 3.94

Table S2. Descriptive statistics of environmental variables measured at successful and depredated nests of water rail (mean ± se). For categorical variables (vegetation species, vegetation stage) the percentage of nests in each category is shown. In vegetation species only three most common species are included.

Variable	Successful nests (*n* = 19)	Depredated nests (*n* = 31)
**Landscape scale**		
Arable land (%)	18.28 ± 4.59	13.48 ± 3.63
Urbanized area (%)	1.13 ± 0.65	1.18 ± 0.42
Emergent vegetation (%)	11.77 ± 1.21	14.22 ± 1.21
Woodie vegetation (%)	3.09 ± 0.81	3.62 ± 0.64
Water body shape (m/ha)	694.9 ± 67.7	613.0 ± 34.9
Water body fragmentation (ha/m)	0.187 ± 0.031	0.236 ± 0.052
**Territory scale**		
Reed cover (%)	18.99 ± 6.12	5.95 ± 1.90
Sedges cover (%)	3.68 ± 3.13	10.61 ± 3.58
Willow cover (%)	10.29 ± 4.35	8.93 ± 2.75
Open water(%)	5.18 ± 2.37	7.45 ± 2.25
Vegetation density (%)	47.24 ± 2.54	33.84 ± 2.11
Water depth (cm)	15.35 ± 1.88	16.96 ± 1.98
**Nest-site scale**		
Vegetation species (*Typha* spp.)	42.1%	45.2%
Vegetation species (*Carex* spp.)	26.3%	35.5
Vegetation species (*Juncus effusus*)	15.8%	6.5%
Vegetation stage (previous years)	42.1%	45.3%
Vegetation stage (mixed)[c]	52.6%	51.6%
Vegetation height (cm)	122.7 ± 9.7	104.4 ± 5.8
Vegetation cover (%)	68.42 ± 3.25	58.39 ± 2.97
Water depth (cm)	16.46 ± 2.33	17.69 ± 1.86
