# Supplementary material for: The adaptive value of habitat preferences from a multi-scale spatial perspective: insights from marsh-nesting avian species
Source: PeerJ. 2017 Mar 28;5:e3164. doi: 10.7717/peerj.3164 (PMC5372843; doi:10.7717/peerj.3164)
Supplement: Supplemental Information 2 [file peerj-05-3164-s002.pdf]

Table S3. Multi-scale glmmPQL models describing the effect of variables selected by GLS models on nest fate. Year and initiation day were treated as fixed variables. Coefficients refer to standardized variables.

Variable	β	SE	*P*
**Little crake**			
intercept	-5.01	0.47	
year (2013)	-0.37	0.47	0.442
year (2014)	-0.52	0.57	0.365
initiation day	-0.50	0.19	0.013
vegetation density	1.55	0.25	<0.001
vegetation height	0.90	0.21	<0.001
water depth (territory)	0.42	0.17	0.017
**Water rail**			
intercept	3.78	0.40	
year (2013)	0.37	0.59	0.537
year (2014)	-0.76	0.45	0.096
initiation day	-0.16	0.21	0.438
emergent vegetation	-0.49	0.15	0.003
vegetation density	0.76	0.21	<0.001
vegetation height	0.29	0.26	0.272
reed cover	0.44	0.30	0.146
